# Supplementary material for: The long non-coding RNA HOTAIR contributes to joint-specific gene expression in rheumatoid arthritis
Source: Nat Commun. 2023 Dec 9;14:8172. doi: 10.1038/s41467-023-44053-w (PMC10710443; doi:10.1038/s41467-023-44053-w)
Supplement: Supplementary file 1 — Supplementary Information [file 41467_2023_44053_MOESM1_ESM.pdf]

The long non-coding RNA HOTAIR plays a major role in joint-specific gene expression in rheumatoid arthritis

Muriel Elhai[1], Raphael Micheroli[1], Miranda Houtman[1], Masoumeh Mirrahimi[1], Larissa Moser[1], Chantal Pauli[2], Kristina Bürki[1] , Andrea Laimbacher[1], Gabriela Kania[1], Kerstin Klein[1,3,4], Philipp Schätzle[5], Mojca Frank Bertoncelj[1], Sam G. Edalat[1], Leandra Keusch[1], Alexandra Khmelevskaya[1], Melpomeni Toitou[1], Celina Geiss[1], Thomas Rauer[6], Maria Sakkou[7,8], George Kollias[7,8], Marietta Armaka[9], Oliver Distler[1], Caroline Ospelt[1*]

[1]Center of Experimental Rheumatology, Department of Rheumatology, University Hospital of Zurich, University of Zurich, Zurich, Switzerland

[2]Institute for Pathology and Molecular Pathology, University Hospital Zurich, Zurich 8091, Switzerland

[3]Department of BioMedical Research, University of Bern, Bern, Switzerland

[4]Department of Rheumatology and Immunology, Inselspital, Bern University Hospital, University of Bern, Bern, Switzerland

[5]Cytometry Facility, University of Zurich, Zurich Switzerland

[6] Department of Trauma Surgery, University Hospital Zurich, Zurich, Switzerland

[7]Institute for Bioinnovation, Biomedical Sciences Research Center (BSRC) ‘Alexander Fleming’, Vari, Greece

[8]Department of Physiology, Medical School, National and Kapodistrian University of Athens, Athens, Greece

[9]Institute for Fundamental Biomedical Research, Biomedical Sciences Research Center "Alexander Fleming", Vari, Greece.

*corresponding author

Supplementary Figure S1: Correlation of the Krenn synovitis score with clinical characteristics and proportion box plots of hand and knee synovial tissues.

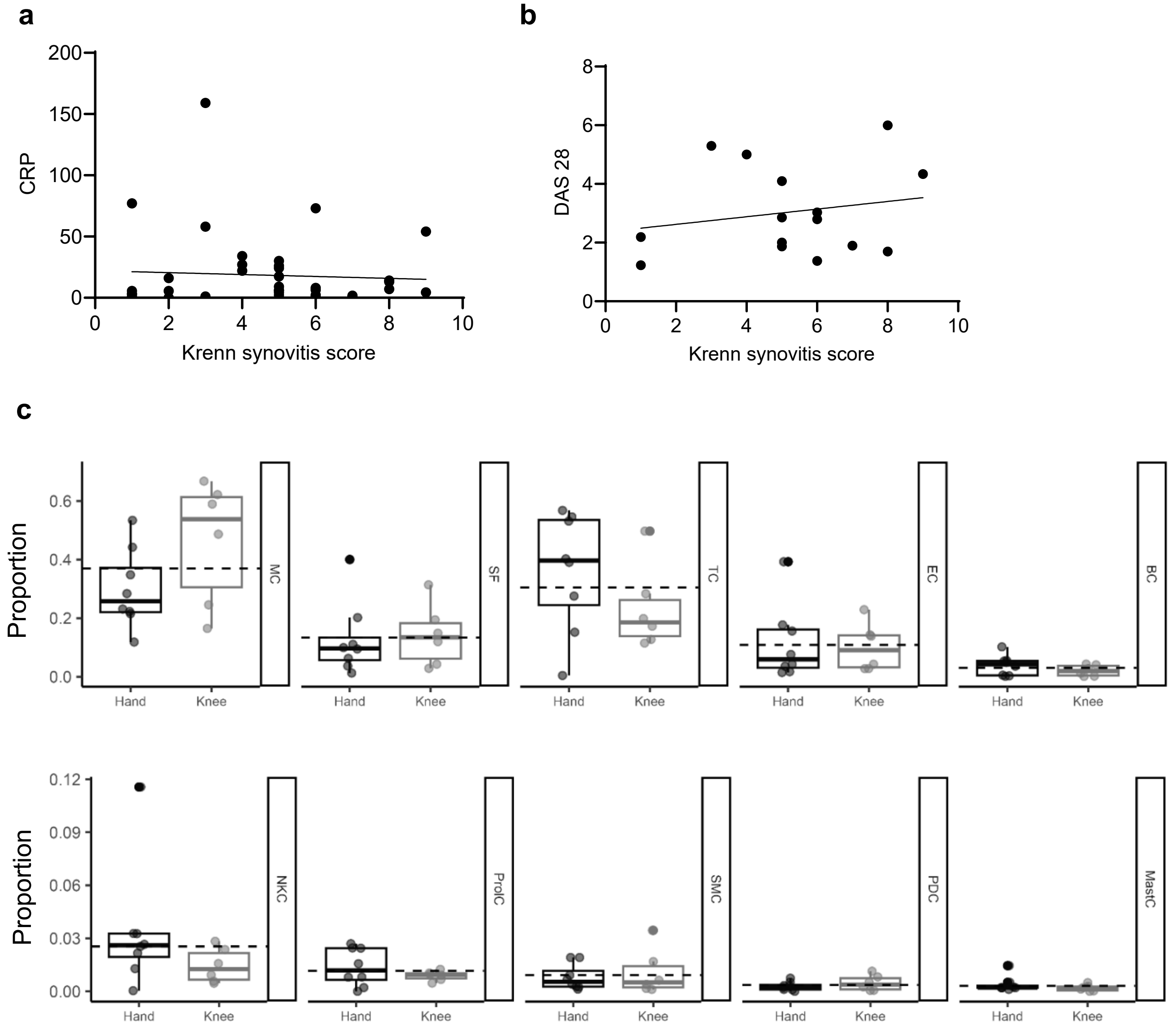

a) Correlation of the Krenn synovitis score with CRP in individual patients (n=40). **b)** Correlation of the Krenn synovitis score with DAS28 in individual patients (n=15). **c)** Boxplots of proportions of individual cell populations as measured with scRNAseq in hand (n=7) and knee (n=6) synovial tissues. MC = myeloid cells, SF = synovial fibroblasts, TC = T cells, EC = endothelial cells, BC = B cells, NKC = natural killer cells, ProlC = proliferating cells, SMC = smooth muscle cells, PDC = plasmacytoid dendritic cells, MastC = mast cells. Min to Max, line at median is shown.

Supplementary Figure S2. HOTAIR silencing in synovial fibroblasts and its effect on H3K27me3 marks.

a

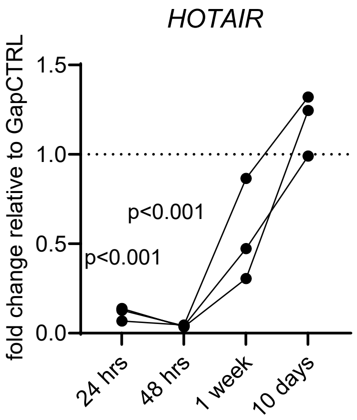

b

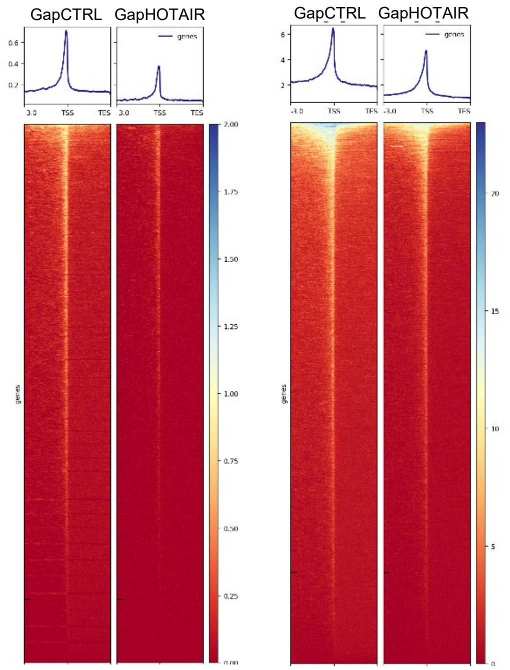

a) Synovial fibroblasts were transfected with control or *HOTAIR* targeting GapmeR for the indicated time points (n=3). One sample t test. **b)** The enrichment of H3K27me3 marks was measured using a CUT&Tag approach in OA synovial fibroblasts transfected with HOTAIR and control GapmeR, respectively (n=2). A heat map and signal profile of enriched H3K27me3 marks 3000bp up- and downstream of the transcription start site (TSS) for each sample is shown.

Supplementary Figure S3. Mechanisms of transcriptional changes induced by *HOTAIR* downregulation.

a

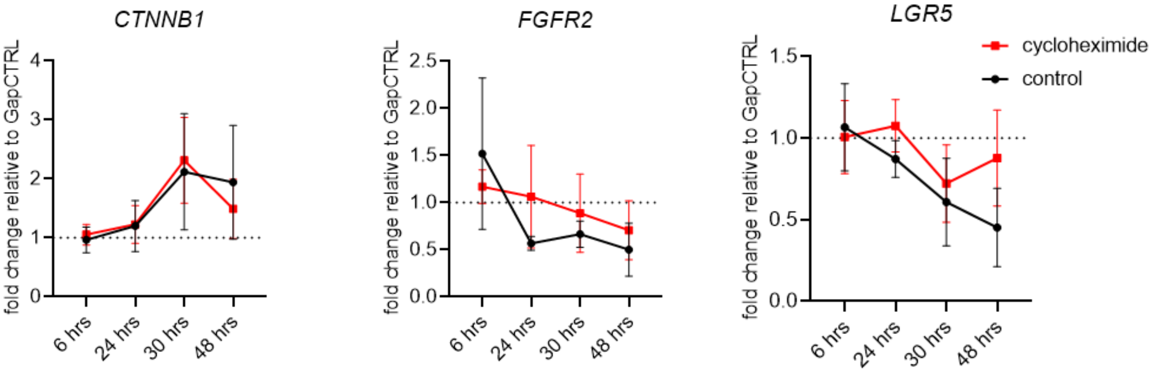

b

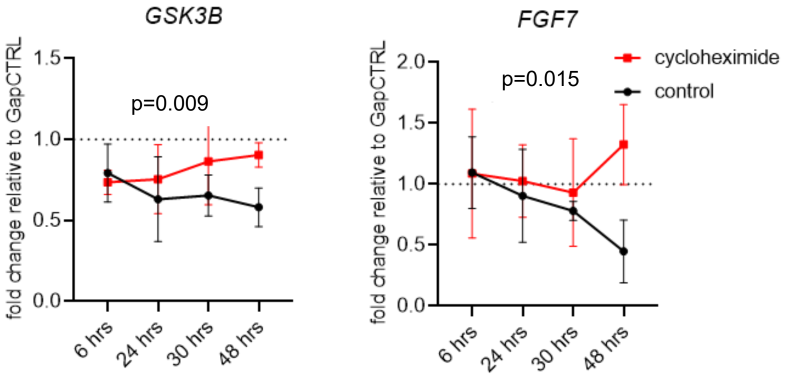

Synovial fibroblasts were transfected with control or *HOTAIR* targeting GapmeR with and without the addition of cycloheximide for 6 hrs (n=5), 24 hrs (n=4), 30 hrs (n=2) and 48 hrs (n=2). The expression of **a)** *CTNNB1, FGFR2* and *LGR5* and of **b)** *GSK3B* and of *FGF7* was measured by qPCR. Mean +/- standard deviation is shown. Two-way ANOVA.

.

Supplementary Figure S4. Changes in *HOTAIR* expression modulate SF subtypes.

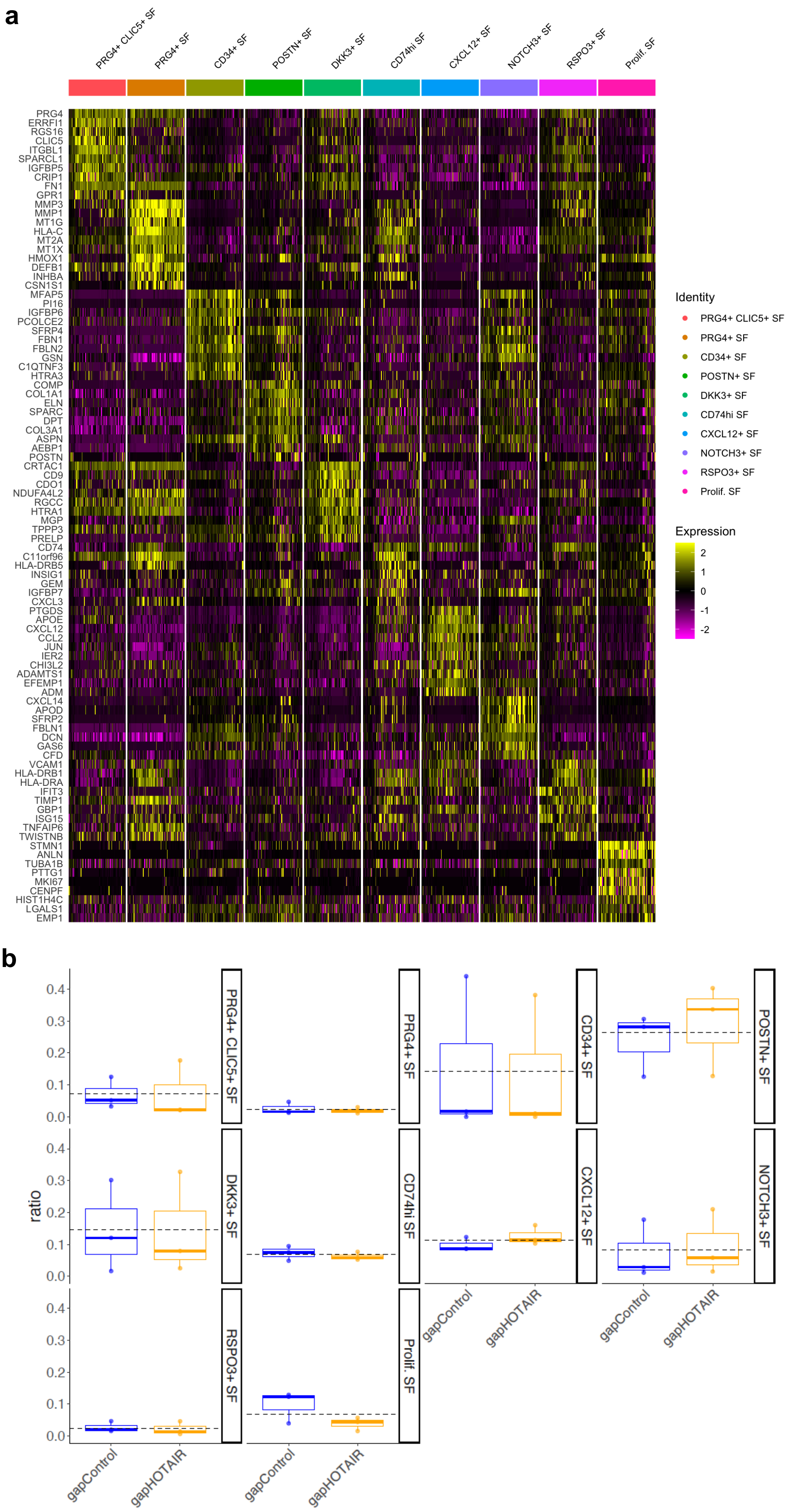

a) Selected marker of gene expression in the different SF subtypes. Heatmap with the top 5 marker genes for each subtype is shown. **b)** Boxplots of proportions of the different cell types in cultured control synovial fibroblasts and *HOTAIR* GapmeR transfected synovial fibroblasts (n=2). Min to Max, line at median is shown.

Supplementary Figure S5. Histological patterns of synovial tissues of OA patients in relation to *HOTAIR* expression levels.

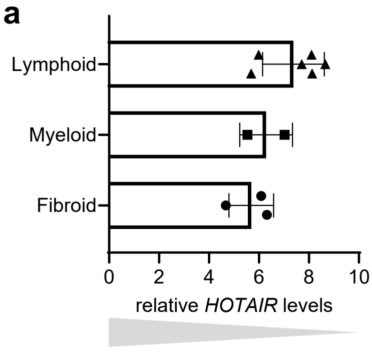

a) Expression levels of *HOTAIR* were measured by qPCR in synovial tissues from osteoarthritis (OA) patients with fibroid/pauci-immune (n=2), myeloid (n=3) or lymphoid (n=6) histological patterns. Mean and range is shown.

Supplementary Figure S6. Gating strategy for measurement of abundance of CD3, CD14 and CD19 positive cells in chemotaxis assay.

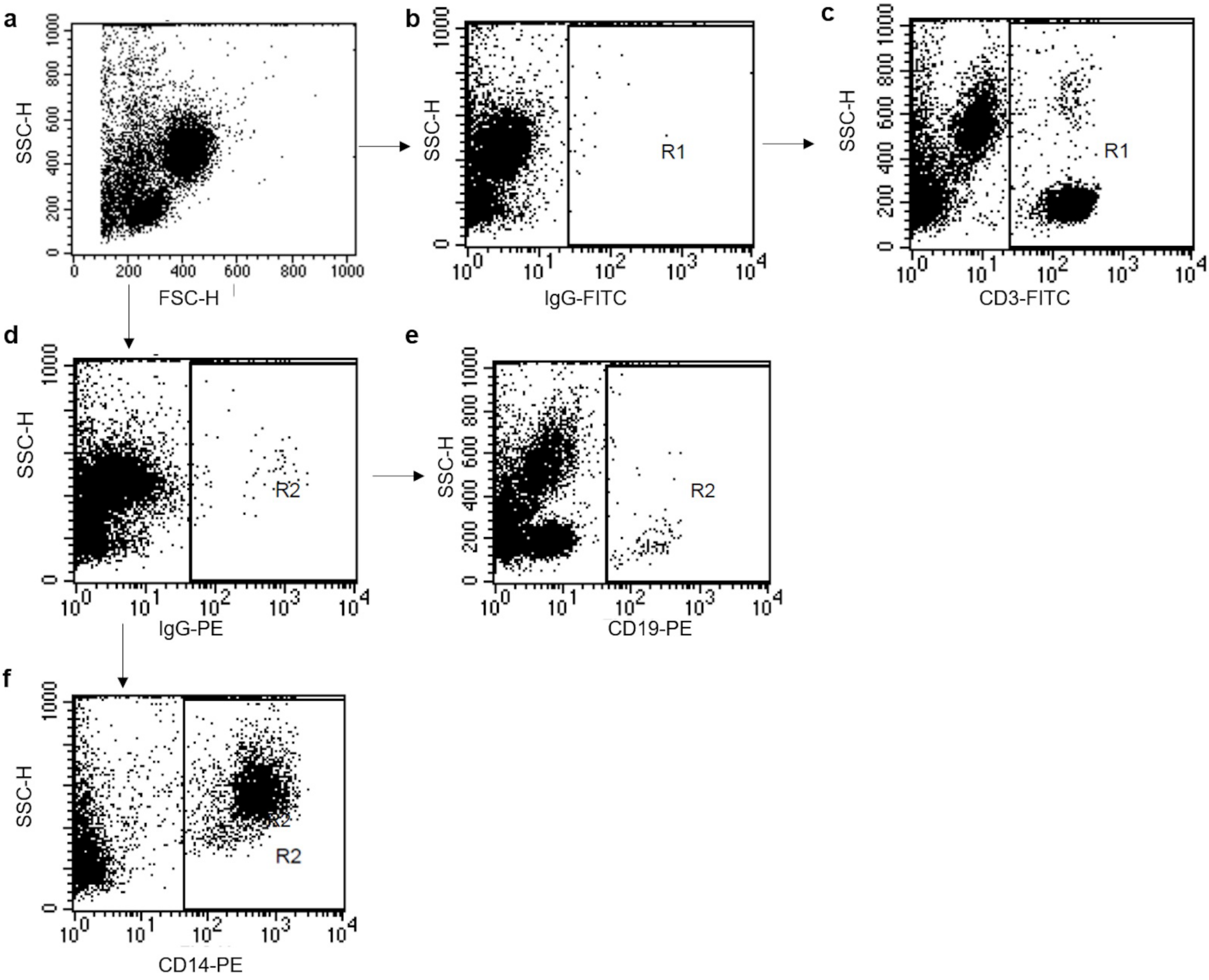

Example of an analysis of PBMCs collected from the lower chamber of a transwell system in the chemotaxis assay. **a)** Forward scatter (FSC-H) and side scatter (SSC-H). **b)** Staining with IgG-FITC (negative control). The gate (R1) was set so that <2% cells were positive. **c)** Staining with CD3-FITC and the gate set in the IgG FITC stained cells. **d)** Staining with IgG-PE (negative control). The gate (R2) was set so that <2% cells were positive. **e)** Staining with CD19-PE or **f)** CD14-PE antibodies and the gates set in the IgG PE stained cells. The percentage of positive cells in the staining minus the percentage in the respective control population was used for the analysis.

Supplementary Table S1: Patient’s characteristics single cell RNA sequencing of RA patients

	Wrist/metacarpophalangeal joint (n=8)	Knee (n=4)
Age (yrs)	59.6 ± 12.0	58.5 ± 19.2
Female (n)	6	4
RF or anti-CCP positivity (n)	5	2 (n=1 with missing information)
Previous biological treatment (n)	7	3 (n=1 with missing information)
Disease modifying treatment at the time of the biopsy (n)	3	0 (n=1 with missing information)

Yrs: years, RF: rheumatoid factor, RA. Rheumatoid arthritis

Supplementary Table S2. Patient's characteristics

In-situ hybridization	OA (n = 11)	RA (n = 9)
Age (yrs)	71.9 ± 7.8	67.9 ± 8.4
Female (n)	7	8
Disease duration (yrs)		22.5 ± 13.8
Knee, hip, feet	11, 0, 0	9, 0, 0
Positive RF		4/5
CRP (mg/L)	1.6 ± 1.3	21.1 ± 26.0
RA treatnent		4 TNF-blocker, 4 steroids, 3 csDMARDS, 1 none
qPCR in synovial tissues	OA (n=12)	RA (N=14)
Age (yrs)	69.0 ± 11.5	69.0 ± 12.2
Female (n)	8	12
Disease duration (yrs)		19.0 ± 12.9
Knee, hip, feet	9, 3, 0	10, 1, 3
Positive RF		100%
CRP (mg/L)	2.4 ± 2.6	11.0 ± 14.7
RA treatnent		5 TNF-blocker, 7 csDMARDS, 2 unknown
qPCR in cultured SF	OA (n=7)	RA (N=8)
Age (yrs)	64 ± 8.2	70 ± 10.5
Female (n)	3	5
Disease duration (yrs)		15 ± 16.3
Knee, hip, feet	4, 3, 0	4, 3,1
Positive RF		83%
CRP (mg/L)	2.6 ± 8.0	2.8 ± 6.4
RA treatnent		1 TNF-blocker, 5 csDMARDS,1 steroid, 1 unknown

OA: osteoarthritis, RA: rheumatoid arthritis, RF: rheumatoid factor, csDMARDS. Conventional disease modifying drugs

Supplementary Table S3. Patient's characteristics (scRNAseq of control SF and SF silenced for HOTAIR) (n=3)

Age (yrs)	72.7 ± 10.2
Female sex (n)	3
Osteoarthritis	3
Knee	3
CRP (mg/L)	1.0 ± 1.0
Treatnent	No

Supplementary Table S4. Sequences of human primers and probes for SybrGreen and TaqMan qPCR measurements

HPRT1	For: 5’ ATG GAC AGG ACT GAA CGT CTT G 3’	Rev:5’ GGC TAC AAT GTG ATG GCC TC 3’
HOTAIR	For: 5’ -GAT CCG AAA GCT TCC ACA GTG-3’	Rev: 5’-AAC TCC CAG GCC TCA GTG C- 3’
HOTAIR Probe	For: 5'-GTG AAA CCA GCC CTA GCC TT-3'	Rev: 5'-GCA GGG TCC CAC TGC ATA AT-3'
TNFα	For: 5’ -TCC AGG CGG TGC TTG TTC-3’	Rev: 5’ -GCC TGC CAC GAT CAG GAA-3’
COL1a1	For: 5’-TCA AGA GAA GGC TCA CGA TGG -3’	Rev: 5’-TCA CGG TCA CGA ACC ACA TT – 3’
COL3a1	For: 5'-TTC CTG GTC TGG CTG GTA CA-3'	Rev: 5'-TCT GAT CCA GGG TTT CCA TCT C-3'
COL1a2	For: 5’- CCG TTG GAC CTC CTG GTA AT-3’	Rev: 5’- CAC CCT TGG CAC CAG TAA GG- 3’
P300	For: 5’– CAG GCA TGG TTC CAG TTT CC-3’	Rev: 5’-CAG GTA GAG GGC CAT TAG AAG TC-3’
CBP	For: 5’-GCA AGC AAA CGG AGA GGT TC-3’	Rev: 5’-ATG CAC AAT GGG CAA CTT GG-3’
FGF2	For: 5’-CTG GCT ATG AAG GAA GAT GGA AG-3’	Rev: 5’- CTC ATC CGT AAC ACA TTT AGA AGC-3’
FGF7	For: 5’- AGC TGT TAG CAA CAA AAC AAA AGT C-3’	Rev: 5’-TCA GTT GCT GTG ACG CTG TT-3’
FGF8	For: 5’- GCT GAG CTG CCT GCT GTT-3’	Rev: 5’- CTG GGC TTG GAG GCA GAG- 3’
FGFR2	For: 5’- GAC CAA ACG TAT CCC CCT GC-3’	Rev: 5’-CTG GAC TCA GCC GAA ACT GTT A-3’
CTNNB1	For: 5’- TTT TGA AAA TCC AGC GTG GAC A-3’	Rev: 5’- CTC CAT CAA ATC AGC TTG AGT AGC-3’
LRP6	For:5’- GTG CTC CTG AGA GCG GC-3’	Rev: 5’- ATC GCA AGT CCC GTC TGT TT- 3’
LGR5	For: 5’- AGA CAC GTA CCC ACA GAA GC-3’	Rev: 5’ - TGG AGA TGT AGG GAT TGA AGG C-3’
GSK3β	For: 5’-TTG GAC TAA GGT CTT CCG ACC CCG-3’	Rev: 5’- CAG TGC AAT TGC CTC CGG TGG A-3’
IL12 A	For: 5’-GCA GTT ATT GAT GAG CTG ATG C-3’	Rev: 5’-GTC TCA CTG TTG AAA TTC AGG G-3’
IL6	For: 5’- CCC TGA GAA AGG AGA CAT GTA AC-3’	Rev: 5’- CCT CTT TGC TGC TTT CAC ACA TG-3’
CXCL12	For: 5’- AGA GCC AAC GTC AAG CAT CT- 3’	Rev: 5’- AGG GCA CAG TTT GGA GTG TT- 3’
PTEN	For : 5’- GCG CTC AGT TCT CTC CTC TC- 3’	Rev : 5’- CAA CTC TCA AAC TTC CAT CAT GG- 3’

FOXO1	For : 5’- ACG AGT GGA TGG TCA AGA GC- 3’	Rev: 5’- GCA CAC GAA TGA ACT TGC TG-3’
RUNX1	For: 5’-ATG CAG GAG GAA GAC ACA GC-3’	Rev: 5’- ACT GAC TTC TGC CTT AAC ATC TCC-3’
Mouse Hotair	For: 5’ CGA CGC CTT CCT TAT AAG CTC 3’	Rev: 5’ CTC TCT CTG CCT TTA CAG TTG C
B2M	For: 5’ CTT CAG CAA GGA CTG GTC TTT C 3’	Rev: 5’ CAT GTC TCG ATC CCA GTA GAC G 3
