# Supplementary material for: The long non-coding RNA HOTAIR contributes to joint-specific gene expression in rheumatoid arthritis
Source: Nat Commun. 2023 Dec 9;14:8172. doi: 10.1038/s41467-023-44053-w (PMC10710443; doi:10.1038/s41467-023-44053-w)
Supplement: Supplementary file 3 — Description of additional supplementary files [file 41467_2023_44053_MOESM3_ESM.docx]

Supplementary Data 1. KEGG 2021 human pathway analysis of genes overexpressed in knee RA

Supplementary Data 2. KEGG 2021 human pathway analysis of genes overexpressed in hand RA

Supplementary Data 3. KEGG pathway analysis of genes changed after HOTAIR silencing in RNA sequencing (FDR < 0.05; LFC +/-1); further analysed pathways are marked in grey

Supplementary Movie 1. Time lapse images of migration of synovial fibroblasts transfected with *HOTAIR* and control GapmeR, respectively over 48 h.
